# Insulin Modulates the Bioenergetic and Thermogenic Capacity of Rat Brown Adipocytes In Vivo by Modulating Mitochondrial Mosaicism

**DOI:** 10.3390/ijms21239204

**Published:** 2020-12-03

**Authors:** Igor Golic, Andjelika Kalezic, Aleksandra Jankovic, Slavica Jonic, Bato Korac, Aleksandra Korac

**Affiliations:** 1 Center for Electron Microscopy, Faculty of Biology, University of Belgrade, 11000 Belgrade, Serbia; igor.golic@bio.bg.ac.rs; 2 Institute for Biological Research “Sinisa Stankovic”—National Institute of Republic of Serbia, University of Belgrade, 11000 Belgrade, Serbia; andjelika.kalezic@ibiss.bg.ac.rs (A.K.); aleksandra.jankovic@ibiss.bg.ac.rs (A.J.); koracb@ibiss.bg.ac.rs (B.K.); 3 IMPMC-UMR CNRS 7590, Sorbonne Université, Muséum National d’Histoire Naturelle, 75005 Paris, France; slavica.jonic@upmc.fr

**Keywords:** brown adipocyte mitochondria, insulin, ETC complexes, ATP synthase, UCP1

## Abstract

The effects of insulin on the bioenergetic and thermogenic capacity of brown adipocyte mitochondria were investigated by focusing on key mitochondrial proteins. Two-month-old male Wistar rats were treated acutely or chronically with a low or high dose of insulin. Acute low insulin dose increased expression of all electron transport chain complexes and complex IV activity, whereas high dose increased complex II expression. Chronic low insulin dose decreased complex I and cyt *c* expression while increasing complex II and IV expression and complex IV activity. Chronic high insulin dose decreased complex II, III, cyt *c*, and increased complex IV expression. Uncoupling protein (UCP) 1 expression was decreased after acute high insulin but increased following chronic insulin treatment. ATP synthase expression was increased after acute and decreased after chronic insulin treatment. Only a high dose of insulin increased ATP synthase activity in acute and decreased it in chronic treatment. ATPase inhibitory factor protein expression was increased in all treated groups. Confocal microscopy showed that key mitochondrial proteins colocalize differently in different mitochondria within a single brown adipocyte, indicating mitochondrial mosaicism. These results suggest that insulin modulates the bioenergetic and thermogenic capacity of rat brown adipocytes in vivo by modulating mitochondrial mosaicism.

## 1. Introduction

Brown adipose tissue (BAT) is a unique mammalian organ involved in maintaining body temperature and regulating body weight and energy balance through non-shivering thermogenesis [1]. Dissipation of excess energy can be induced by cold or diet [1,2], and brown adipocytes containing a large number of mitochondria perform this function.

Numerous studies have shown the importance of insulin in the regulation of BAT activity [3,4,5], glucose homeostasis, and insulin sensitivity [6,7]. The interplay between thermogenesis, insulin, and BAT mitochondria and their role in diabetes and obesity has been debated for many years since early studies revealed a specific decrease in mitochondrial thermogenic capacity [8] and major thermogenic defects associated with insulin resistance in brown adipose tissue of obese diabetic rats [9]. It was also shown that mitochondrial dysfunction has a cell type-dependent divergent effect on insulin action [10].

Mitochondria are essential for life and normal cellular function, such as ATP production, calcium homeostasis, synthesis of phospholipids and heme, β-oxidation of fatty acids, activation of apoptosis, and cell death. The inner mitochondrial membrane harbors the major electron transport chain (ETC) complexes I-IV, as well as ATP synthase that utilizes the proton gradient generated by the electron flow through the ETC to produce ATP [11]. Upon BAT activation, uncoupling protein (UCP) 1 inserts into the inner mitochondrial membrane and uncouples respiration and ATP production, dissipating the proton gradient and producing heat [12].

Regarding UCP1 presence in BAT, Cinti et al. [13] reported that after cold acclimation or administration of a β_3_-receptor agonist, BAT displays heterogeneous UCP1 expression known as the Harlequin effect, which indicates heterogeneity in the thermogenic capacity of brown adipocytes. It has been proposed that the role of Harlequinism might be the result of alternative UCP1 gene activation and protein expression in neighboring brown adipocytes as a kind of protective mechanism to avoid metabolic and thermogenic damage in stimulated brown adipocytes [13]. Although brown adipocytes also show intracellular mitochondrial heterogeneity, e.g., differences between peridroplet and cytoplasmic mitochondria [14], the exact role of the Harlequin effect remains unclear. Particularly, it is not known whether brown adipocytes exhibit heterogeneous expression of other mitochondrial proteins, especially those involved in bioenergetic capacity. Therefore, there is a need to investigate BAT mitochondria metabolism in a subpopulation-specific manner and in the presence of physiological stimuli that modify the mitochondrial function in vivo.

Mitochondrial energy-transducing capacity is essential for the maintenance of cellular function, and impaired mitochondrial energy metabolism/redox homeostasis is a hallmark of metabolic diseases such as obesity and diabetes [15,16,17]. Mitochondria are organized in networks by fission and fusion events, and both heterogeneity and compartmentalization of energy production are visible within mitochondrial tubules, at least in vitro [18], although in vivo data are missing.

The association between mitochondrial homeostasis and insulin signaling has received much attention recently. A study on liver mitochondria in diabetic and non-diabetic rats revealed that insulin is important for efficient ATP production [19], and recent studies on skeletal muscle showed that insulin improves mitochondrial function [20,21]. However, Boirie et al. [22] reported that insulin affects mitochondrial proteins in a tissue-specific manner, and insulin is reported to induce insulin resistance more than hyperglycemia, as shown in diabetes type 1 [23]. Mild hyperinsulinemia can be tolerated for a short time, but chronic hyperinsulinemia triggers insulin resistance and contributes to the etiology of diabetes [24]. The role of mitochondria in insulin resistance is still controversial, and it is not clear whether mitochondrial dysfunction is a cause or consequence of insulin resistance, at least in skeletal muscle [16,25].

Our previous studies also showed that insulin treatment affects BAT mitochondria, were a high chronic dose induced mitochondrial damage and lipofuscin formation [26] as well as apoptosis of brown adipocytes [27], demonstrating the role of insulin in tissue energetic/thermogenic profiling.

In this study, we attempted to elucidate how insulin modulates the mitochondrial bioenergetic and thermogenic capacity of brown adipocytes by analyzing the expression and colocalization of key mitochondrial proteins, i.e., ETC complexes, ATP synthase, and UCP1.

## 2. Results

### 2.1. Transmission Electron Microscopy

Bearing in mind that the protein expression profile in whole tissue does not represent mitochondria-only proteins, we first decided to examine the effects of hyperinsulinemia on key mitochondrial ETC complexes, ATP synthase, and UCP1 protein expression on isolated mitochondria. In order to check the yield, purity, and quality of mitochondrial isolation, we used transmission electron microscopy.

**Figure 1:**
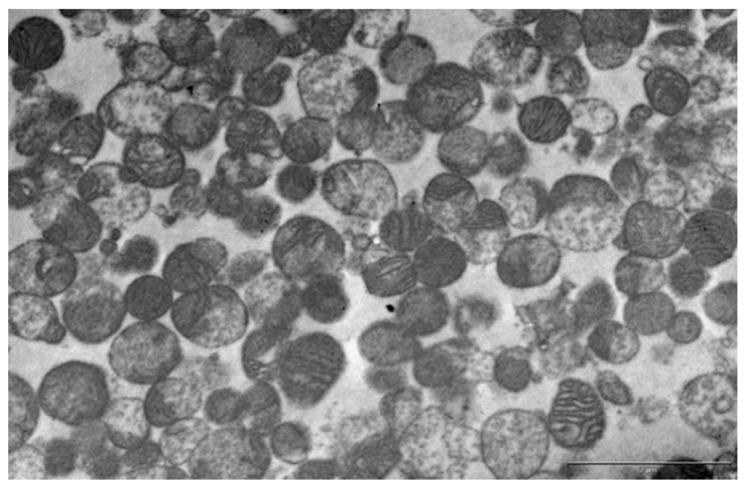
Representative transmission electron microscopy of the mitochondria-enriched fraction from brown adipose tissue (BAT) of acute low (0.4 IU) dose insulin-treated rats. Scale bar = 2 µm.

Careful examination of randomly chosen samples (pooled from six animals in each group; 18 images per group) confirmed that mitochondria-enriched fractions (MEF) were devoid of cellular contaminants (i.e., the nucleus, microsomes, or plasma membrane) and consisted of rounded mitochondria with a variable number of lamellar cristae similar in size and appearance to those from brown adipocytes (Figure 1).

### 2.2. Expression of Electron Transport Chain Components in MEF

**Figure 2:**
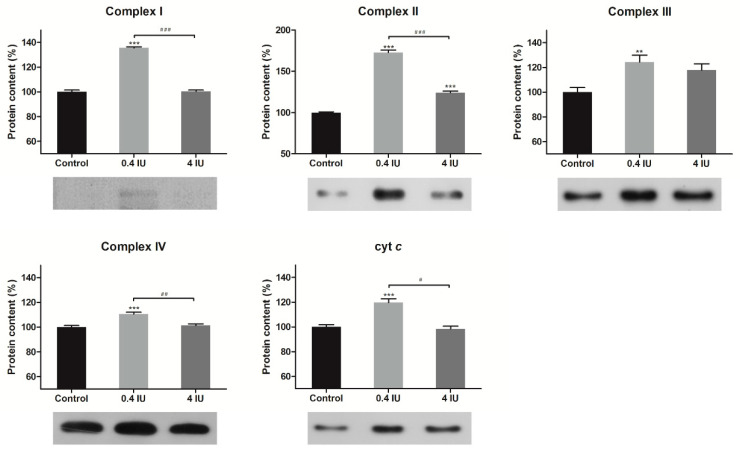
Effects of acute insulin treatment on the expression of electron transport chain (ETC) complexes and cyt *c* in mitochondria-enriched fractions (MEF). Protein content is expressed relative to controls, which were standardized to 100%. Mean ± SEM values are averages of three independent experiments performed in triplicate (pooled from six animals in each group). A representative blot is shown. Volume is the sum of all pixel intensities within a band (1 pixel = 0.007744 mm^2^). * compared to control: ** *p* ≤ 0.01, *** *p* ≤ 0.001; ^#^ 0.4 IU vs. 4 IU: ^#^ *p* ≤ 0.05, ^##^ *p* ≤ 0.01, ^###^ *p* ≤ 0.001.

**Figure 3:**
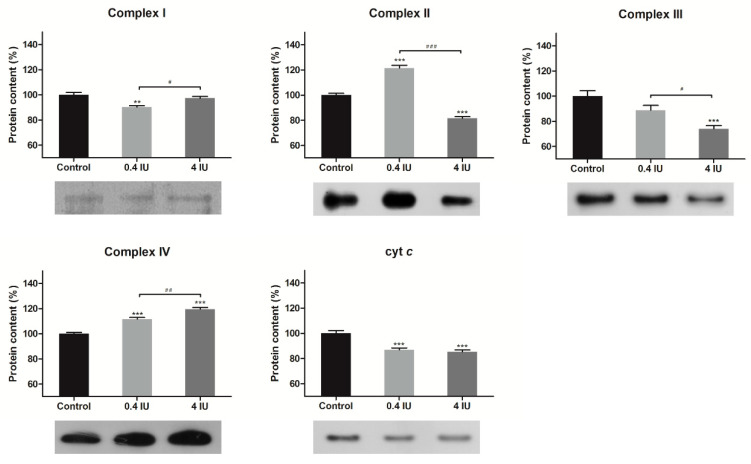
Effects of chronic insulin treatment on the expression of ETC complexes and cyt *c* in MEF. Protein content is expressed relative to controls, which were standardized to 100%. Mean ± SEM values are averages of three independent experiments performed in triplicate (pooled from six animals in each group). A representative blot is shown. Volume is the sum of all pixel intensities within a band (1 pixel = 0.007744 mm^2^). * compared to control: ** *p* ≤ 0.01, *** *p* ≤ 0.001; ^#^ 0.4 IU vs. 4 IU: ^#^ *p* ≤ 0.05; ^##^ *p* ≤ 0.01; ^###^ *p* ≤ 0.001.

The protein expression levels of the ETC complexes in the BAT mitochondria-enriched fractions were determined by Western Blot analysis following acute (Figure 2) or chronic (Figure 3) insulin treatment with either a low (0.4 IU) or high (4 IU) dose, respectively.

Acute treatment with a low dose of insulin increased protein expression of all examined ETC complexes and cyt *c*, while acute high dose of insulin increased only complex II protein level, compared to control.

Compared to control, a chronic low dose of insulin decreased protein expression of complex I and cyt *c*, and increased complex II and IV expression. Chronic high dose of insulin decreased the protein expression of complex II, III, and cyt *c*, and increased complex IV expression.

### 2.3. UCP1 Expression in MEF

**Figure 4:**
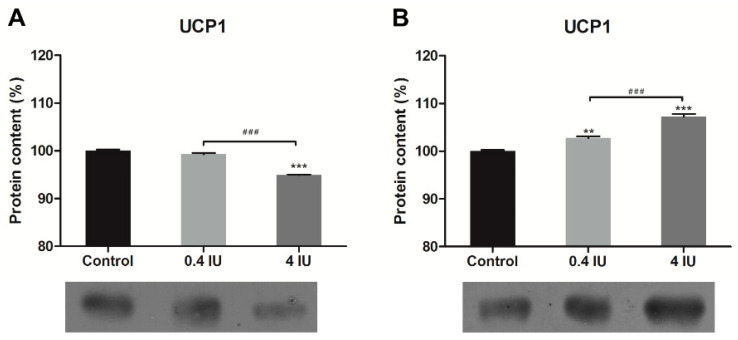
Effects of acute (**A**) and chronic (**B**) insulin treatment on the expression of uncoupling protein (UCP) 1 in MEF. Protein content is expressed relative to controls, which were standardized to 100%. Mean ± SEM values are averages of three independent experiments performed in triplicate (pooled from six animals in each group). A representative blot is shown. Volume is the sum of all pixel intensities within a band (1 pixel = 0.007744 mm^2^). * compared to control: ** *p* ≤ 0.01, *** *p* ≤ 0.001; ^#^ 0.4 IU vs. 4 IU: ^###^ *p* ≤ 0.001.

In comparison to control, acute insulin treatment decreased UCP1 protein expression in the high (4 IU) insulin group (Figure 4A). In contrast, both doses of chronic insulin treatment increased UCP1 protein expression (Figure 4B).

### 2.4. Expression of ATP Synthase and IF1 in MEF

**Figure 5:**
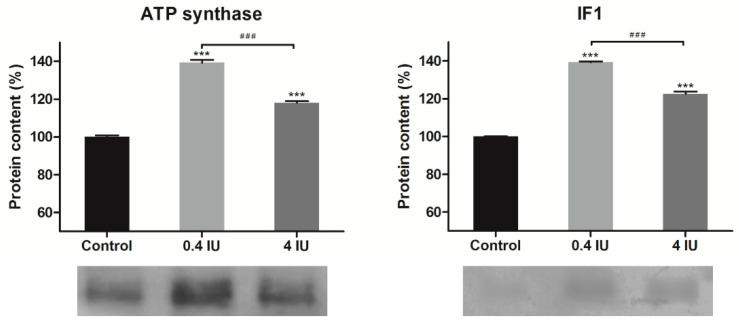
Effects of acute insulin treatment on the expression of ATP synthase and inhibitory factor 1 (IF1) in MEF. Protein content is expressed relative to controls, which were standardized to 100%. Mean ± SEM values are averages of three independent experiments performed in triplicate (pooled from six animals in each group). A representative blot is shown. Volume is the sum of all pixel intensities within a band (1 pixel = 0.007744 mm^2^). * compared to control: *** *p* ≤ 0.001; ^#^ 0.4 IU vs. 4 IU: ^###^ *p* ≤ 0.001.

**Figure 6:**
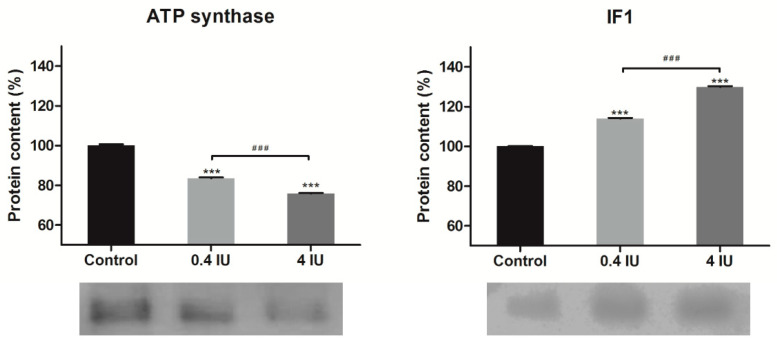
Effects of chronic insulin treatment on the expression of ATP synthase and IF1 in MEF. Protein content is expressed relative to controls, which were standardized to 100%. Mean ± SEM values are averages of three independent experiments performed in triplicate (pooled from six animals in each group). A representative blot is shown. Volume is the sum of all pixel intensities within a band (1 pixel = 0.007744 mm^2^). * compared to control: *** *p* ≤ 0.001; ^#^ 0.4 IU vs. 4 IU: ^###^ *p* ≤ 0.001.

Acute insulin treatment increased ATP synthase and ATPase inhibitory factor 1 (IF1) protein expression, independent of the applied dose (Figure 5). In contrast, chronic insulin treatment decreased ATP synthase but increased IF1 levels, and this effect was more prominent in the high (4 IU) dose group (Figure 6).

### 2.5. Complex IV and ATP Synthase Activity

To further investigate whether insulin-induced changes of complex IV and ATP synthase protein expression levels also affect their function, we determined complex IV and ATP synthase activity in BAT mitochondria-enriched fractions following acute or chronic treatment with either low (0.4 IU) or high (4 IU) insulin dose, respectively.

**Figure 7:**
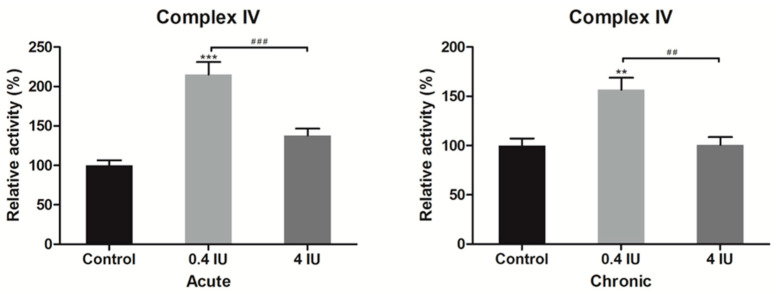
Relative complex IV activity following acute and chronic insulin treatment. Data showing complex IV activity, expressed relative to a control (taken as 100%), represent the means ± SEM values of three independent experiments. * compared to control: ** *p* ≤ 0.01, *** *p* ≤ 0.001; ^#^ 0.4 IU vs. 4 IU: ^##^ *p* ≤ 0.01, ^###^ *p* ≤ 0.001.

Acute and chronic treatments with a low dose of insulin increased complex IV activity compared to control (Figure 7).

**Figure 8:**
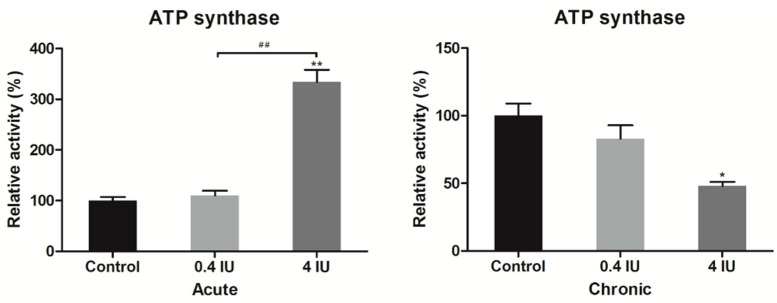
Relative ATP synthase activity following acute and chronic insulin treatment. Data showing ATP synthase activity, expressed relative to a control (taken as 100%), represent the means ± SEM values of three independent experiments. * compared to control: * *p* ≤ 0.05, ** *p* ≤ 0.01; ^#^ 0.4 IU vs. 4 IU: ^##^ *p* ≤ 0.01.

Acute insulin treatment increased, while chronic insulin treatment decreased ATP synthase activity only in high (4 IU) dose treated groups (Figure 8).

### 2.6. Mitochondrial Protein Stoichiometry

Immunoblotting showed that insulin affected protein expression differently, so we further calculated their ratio. The ratio of interest is ATP synthase:IF1 stoichiometry (Table 1)—both doses of insulin decreased this ratio in acute-treated groups, where a low dose of insulin showed a more prominent effect. A similar pattern was observed with ATP synthase:UCP1 stoichiometry.

Chronic insulin administration increased ATP synthase:IF1 and ATP synthase:UCP1 stoichiometry ratios in both dosage treatments. It should be pointed out that the UCP1:IF1 ratio was somewhat stable during insulin treatment.

### 2.7. Immunofluorescence of ETC Complexes and UCP1

**Figure 9:**
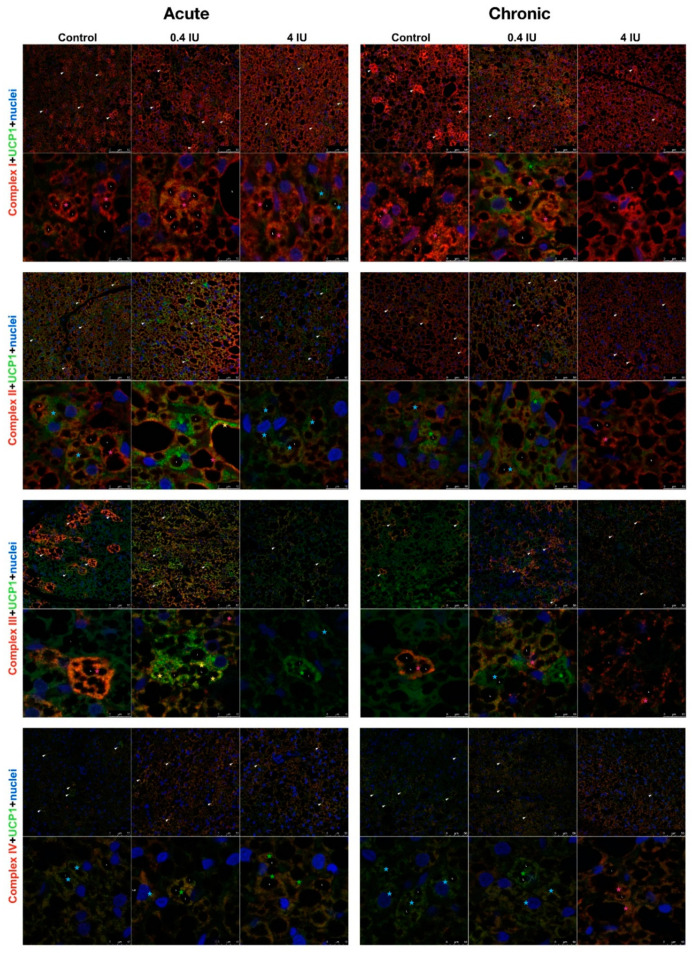
Immunofluorescence of UCP1 and ETC complexes in BAT following acute and chronic insulin treatment. Double labeling with antibodies against UCP1 (green) and ETC complexes (red). Harlequin effect is shown with arrowheads. BAT showed various brown adipocytes with different degree of immunoexpression: UCP1-only brown adipocytes (light-blue star); UCP1 predominantly positive brown adipocytes (green star); brown adipocytes with clear colocalization of UCP1 and specific ETC complex (yellow star); specific ETC complex predominantly positive brown adipocytes (pink star); and specific ETC complex-only brown adipocytes (red star). L—lipid bodies. Nuclei were stained with Sytox Orange. Scale bar = 50 µm (first row) and 10 µm (second row).

Considering these clear and prominent insulin-induced changes of bioenergetic and thermogenic proteins, we analyzed whether this effect was related to the Harlequin pattern of UCP1 localization in BAT. Therefore, cellular localization and immunoexpression of ETC complexes and UCP1 were studied by confocal microscopy following acute or chronic treatment with a low (0.4 IU) or high (4 IU) dose of insulin (Figure 9).

In all experimental groups, brown adipocytes showed a clear heterogeneous immunolocalization of different ETC complexes and UCP1, known as the Harlequin effect (Figure 9). Several types of brown adipocytes were observed based on UCP1 and specific ETC complex immunoexpression (Figure 9). Some were only or predominantly positive for UCP1 or ETC complex, some were UCP1 negative (e.g., preadipocytes), while others displayed clear colocalization of UCP1 and specific ETC complexes. Individual white adipocyte-like adipocytes were interspersed within BAT and exhibited strong immunolocalization of complexes I, II, and III around lipid bodies (L).

**Figure 10:**
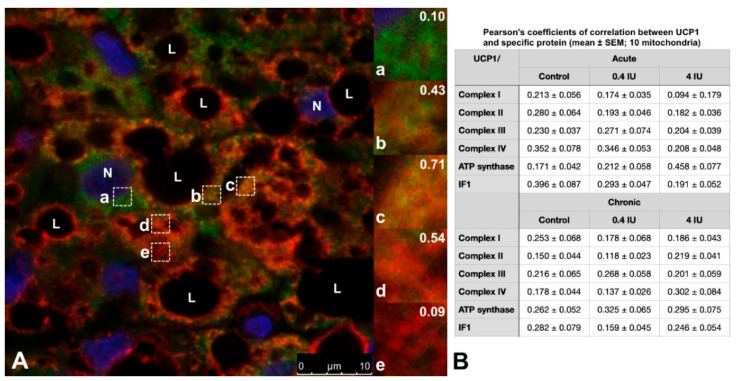
Representative images showing the Harlequin effect in brown adipocyte and the types of mitochondria. Double labeling with antibodies against UCP1 (green) and complex I (red). Nuclei were stained with Sytox Orange. (**A**) Representative BAT image from chronic low (0.4 IU) insulin group. Brown adipocytes showed various mitochondrial types: a. UCP1-only positive mitochondria; b. UCP1 predominantly positive mitochondria; c. UCP1 and complex I colocalized mitochondria; d. complex I predominantly positive mitochondria; e. complex I-only positive mitochondria. Pearson’s coefficient of correlation is shown in the upper right corner for each type of mitochondria. (**B**) Pearson’s coefficients of correlation between UCP1 and specific protein (mean ± SEM; 10 mitochondria within single brown adipocyte from each group). Scale bar = 10 µm (**A**).

Moreover, brown adipocytes showed immunofluorescence heterogeneity at the mitochondrial level (Figure 10A). The mitochondrial heterogeneity was confirmed by analyzing Pearson’s coefficient of correlation between UCP1 and mitochondrial proteins of interest, performed on ten mitochondria within a single brown adipocyte in all experimental groups (Figure 10B).

Smaller and less mature brown adipocytes displayed lower UCP1 fluorescence compared to the adjacent larger and mature brown adipocytes. Brown adipocytes with numerous and smaller lipid bodies, exhibited stronger UCP1 immunofluorescence, too. In contrast, complex I-positive mitochondria were more intense around lipid bodies in brown adipocytes with bigger lipid bodies. In acute groups, complex I and UCP1 fluorescence in the high (4 IU) insulin group was reduced compared to the low (0.4 IU) insulin group and control. In chronically treated groups, complex I immunofluorescence appeared at a similar level. Complex I immunofluorescence is more localized at the BAT periphery in both controls. Complex II shared a similar immunoexpression pattern as complex I. Complex II positive brown adipocytes were randomly dispersed and more localized near fibrous septa in both controls.

We also found a mosaic pattern of brown adipose cells exhibiting various levels of UCP1 and complex III localization, where more complex III immunofluorescence was observed in the acute low (0.4 IU) insulin group.

Compared to other ETC complexes, more colocalization of complex IV and UCP1 was observed in brown adipocytes in insulin-treated groups. Complex IV positive brown adipocytes were mostly localized at the BAT periphery in all experimental groups.

### 2.8. Immunofluorescence of ATP Synthase/UCP1 and IF1/UCP1

**Figure 11:**
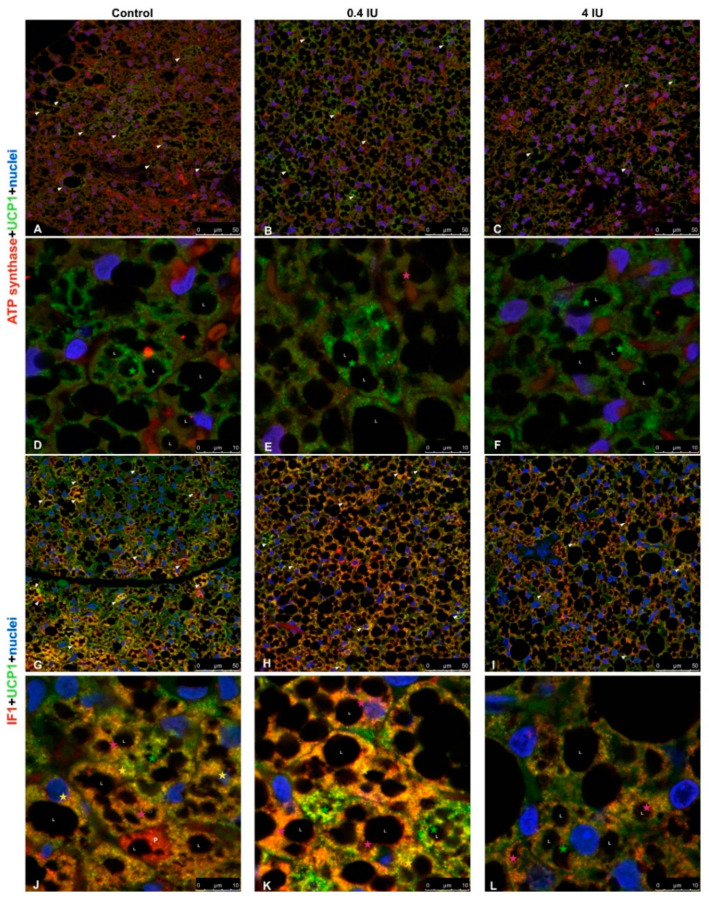
Immunofluorescence of UCP1/ATP synthase and UCP1/IF1 in BAT following acute insulin treatment. (**A**–**F**) Double labeling with antibodies against UCP1 (green) and ATP synthase (red). (**G**–**L**) Double labeling with antibodies against UCP1 (green) and IF1 (red). Harlequin effect is shown with arrowheads. BAT showed various brown adipocytes with different degrees of immunoexpression: UCP1-only brown adipocytes (light-blue star); UCP1 predominantly positive brown adipocytes (green star); brown adipocytes with clear colocalization of UCP1 and ATP synthase/IF1 (yellow star); ATP synthase/IF1 predominantly positive brown adipocytes (pink star); and ATP synthase/IF1-only brown adipocytes (red star). L—lipid body; P—preadipocyte. Nuclei were stained with Sytox Orange. (**A**–**C**,**G**–**I**), scale bar = 50 µm. (**D**–**F**,**J**–**L**), scale bar = 10 µm.

Confocal microscopy of brown adipocytes from acutely low (0.4 IU) and high (4 IU) dose insulin-treated animals showed heterogeneity of UCP1 and ATP synthase immunofluorescence, as did the control group (Figure 11A–C). Both control and insulin-treated groups showed that the majority of UCP1 positive mitochondria were localized around lipid bodies (L), while ATP synthase positive mitochondria mostly remained in the cytoplasm of brown adipocytes after acute insulin treatments (Figure 11D–F).

Colocalization studies using anti-UCP1 and anti-ATPase inhibitory factor 1 (IF1) primary antibodies revealed heterogeneity of UCP1 or IF1 localization brown adipocytes in the control group (Figure 11G). That randomly dispersed Harlequin pattern of UCP1 and/or IF1 positive brown adipocytes was diminished in acutely treated groups, and overall IF1 immunofluorescence was higher in the low (0.4 IU) dose group compared to the high (4 IU) dose group and control (Figure 11H,I). Preadipocytes did not show UCP1 immunofluorescence (Figure 11J), while brown adipocytes displayed a range of UCP1 and IF1 (co)localization in mitochondria (Figure 11K,L).

**Figure 12:**
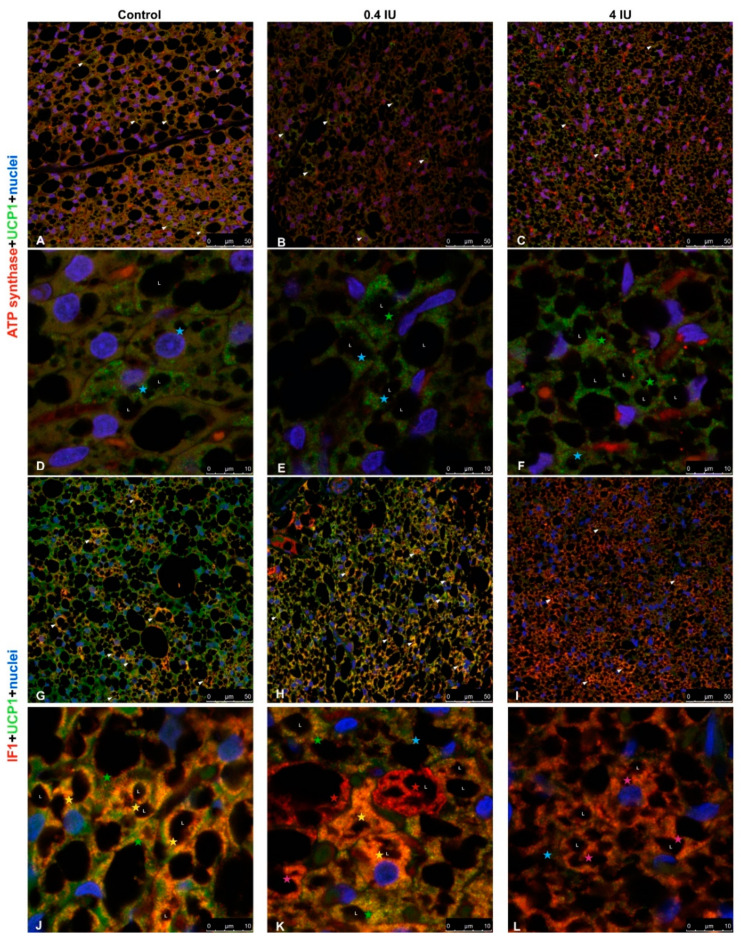
Immunofluorescence of UCP1/ATP synthase and UCP1/IF1 in BAT following chronic insulin treatment. (**A**–**F**) Double labeling with antibodies against UCP1 (green) and ATP synthase (red). (**G**–**L**) Double labeling with antibodies against UCP1 (green) and IF1 (red). Harlequin effect is shown with arrowheads. BAT showed various brown adipocytes with different degrees of immunoexpression: UCP1-only brown adipocytes (light-blue star); UCP1 predominantly positive brown adipocytes (green star); brown adipocytes with clear colocalization of UCP1 and ATP synthase/IF1 (yellow star); ATP synthase/IF1 predominantly positive brown adipocytes (pink star); and ATP synthase/IF1-only brown adipocytes (red star). L—lipid body. Nuclei were stained with Sytox Orange. (**A**–**C**,**G**–**I**), scale bar = 50 µm. (**D**–**F**,**J**–**L**), scale bar = 10 µm.

In chronically treated animals, similar heterogeneity and localization of UCP1 and ATP synthase immunoexpression in BAT were revealed, as in acute groups, but ATP synthase immunofluorescence was diminished in both chronically treated groups compared to control (Figure 12A–F). The gradual dose-dependent increase of IF1 immunofluorescence was observed in insulin-treated groups compared to control (Figure 12G–L).

**Figure 13:**
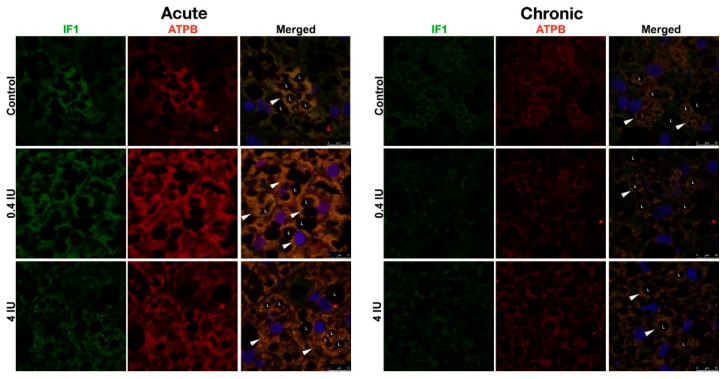
Immunofluorescence of IF1/ATP synthase in BAT following acute and chronic insulin treatment. Double labeling with antibodies against IF1 (green) and ATP synthase (red). Harlequin effect is shown with arrowheads. L—lipid body. Nuclei were stained with Sytox Orange. Scale bar = 10 µm.

Furthermore, double immunofluorescence using anti-IF1 and anti-ATP synthase primary antibodies (Figure 13) revealed their strong colocalization, independent of the applied dose.

## 3. Discussion

In the present study, we have examined the effects of insulin on crucial proteins involved in brown adipocytes’ mitochondria bioenergetic and thermogenic capacity by analyzing the expression, activity, and immunolocalization of key mitochondrial proteins. Rats were treated either acutely or chronically with low (0.4 IU) or high (4 IU) doses of insulin. We observed a number of prominent effects which proved to be dose- and treatment duration-dependent. The main finding was that insulin modulates the bioenergetic and thermogenic capacity of rat brown adipocytes in vivo by modulating mitochondrial mosaicism.

We found that acute insulin treatment stimulates protein expression of all studied ETC complexes in a low dose, whereas a high dose stimulates only complex II expression. This is a consequence of insulin-stimulated glucose uptake, well documented in vitro [28] and in vivo, in rodent [29] and human BAT [6], which leads to increased acetyl-CoA. Acetyl-CoA enters the citric acid cycle, leading to elevated levels of NADH and succinate, substrates of complex I and complex II, thus enhancing ETC activity.

In the case of chronic insulin treatment, we found that a low dose decreases cyt *c* and complex I protein expression, while a high dose decreases cyt *c*, complex II, and complex III expression. It is possible that chronic glucose uptake leads to excessive NADH production, thereby increasing the mitochondrial proton gradient, and as a result, single electrons are transferred to oxygen, forming reactive oxygen species (ROS) [30]. Under such pressure, cells may reduce ROS production by (a) suppressing NADH production or (b) stopping substrate entry into mitochondria [31]. Insulin also stimulates H_2_O_2_ production, a molecule with an important role in insulin signaling [32,33,34]. In mitochondria respiring on succinate, the bulk of ROS is produced by complex II [35] and complex I during reverse electron transport from complex II [36,37]. In vitro study on neurons showed that insulin-stimulated H_2_O_2_ production enables insulin receptor autophosphorylation in an all-or-nothing manner resulting in increased receptor tyrosine kinase activity [38]. Taken together, insulin stimulates brown adipocytes and regulates protein expression of complex I and complex II to maintain optimal utilization of substrates, balancing between maximizing ATP production and minimizing excessive ROS production, which could be the case in hyperinsulinemia.

Interestingly, complex IV protein level was increased with a low (0.4 IU) dose in both acute- and chronic-treated groups, along with their activity. To the best of our knowledge, data on complex IV expression and activity following insulin treatment in brown adipocytes have not been reported previously, and this is the first study to broach this subject. The fact that a low dose of insulin synergistically enhanced both expression and activity of complex IV underlines their importance for the respiratory chain function since it catalyzes the terminal reaction, four-electron reduction of oxygen to water.

Acute insulin treatment also stimulated ATP synthase expression which is consistent with the observed increase in ETC complexes expression. This would be expected to lead to the translocation of a greater number of protons through the inner mitochondrial membrane and elevated ATP synthase activity following coupling to the proton gradient. Furthermore, chronic insulin treatment decreased ATP synthase expression, along with other components of the ETC, which may reflect a reduction in the efficiency of proton translocation by other components of the ETC. Alternatively, a decrease in ATP synthase expression is reported to be accompanied by an increase in UCP1 expression in BAT, especially during cold acclimation of mice, or in BAT ontogenesis [39], where brown adipocyte fulfills its essential role—using proton gradient to generate heat for maintaining body temperature.

Another intriguing finding from our study is the increased expression of IF1, irrespective of the insulin dose or treatment duration, showing that IF1 expression is under direct insulin control. It opens many questions regarding their role in brown adipocytes mitochondria since we found that chronic insulin treatment, regardless of the dose applied, induced their uncoupling.

Namely, it was previously shown that IF1 inhibits the transition from ATP synthase to ATPase activity in the presence of particular chemical uncouplers such as carbonylcyanide-*p*-trifluoromethoxyphenylhydrazone (FCCP) and 2,4-dinitrophenol (DNP) [40]. Furthermore, proteomic analysis of murine BAT following cold treatment showed a two-fold increase in IF1 expression, and IF1 up-regulation may serve to down-regulate ATP hydrolysis in brown adipocytes mitochondria with an elevated thermogenic capacity [41]. Bearing in mind our finding that chronic insulin treatment stimulated uncoupling, it is possible that IF1 in our study has an additional role, perhaps in maintaining cristae structure, which would be of great importance for brown adipocytes function in particular. So far, Campanella et al. [42] proposed that IF1 has a structural role and contributes to the preservation of the inner mitochondrial membrane structure since it has been reported to stabilize oligomers of ATP synthase and thus determine cristae shapes [43].

It has also been shown that in osteosarcoma cells, IF1 can increase the oxidative phosphorylation rate by improving the structure of mitochondrial cristae or by stimulating the activity of ATP synthase [44]. Thus, elevated ATP synthase activity after acute high-dose insulin treatment compared with ATP synthase protein expression after acute low-dose insulin is not merely due to increased ATP protein expression but due to enhanced activity by IF1. Chronic treatment with a high dose of insulin stimulates metabolic reprogramming, e.g., lipid and glycogen deposition in brown adipocytes (not shown here), leading to decreased ATP synthase activity. The observed parallel and inverse changes in the expression of ATP synthase and the inhibitor IF1 upon acute and chronic insulin treatment, respectively, are of particular interest. To prevent excessive ROS production and unnecessary ATP consumption that would otherwise lead to mitochondrial/brown adipocyte damage, IF1 is colocalized with ATP synthase, as shown earlier in acute-treated groups. However, in chronic-treated groups with a high (4 IU) dose of insulin, IF1 counteract is not successful in preventing mitochondrial peroxidative damage [26] and results in brown adipocytes apoptosis reported earlier by our group [27]. This is consistent with the found decreased expression of cyt *c* after chronic treatment, irrespective of the dose applied.

Observed changes in the protein expression of ETC complexes following insulin treatment may be a result of compensatory mechanisms between different mitochondrial respiratory complexes [45,46,47]. It suggests that acute and chronic insulin treatment modulates the expression of major mitochondrial proteins through fine metabolic reprogramming between mitochondrial thermogenic (uncoupling) and bioenergetic capacity in brown adipocytes. Thus, insulin could promote balancing between the bioenergetic and thermogenic phenotype of brown adipocyte by modulating its signature molecules, e.g., respiratory chain complexes, IF1, and UCP1. Whether variations in mitochondrial complexes expression contribute to metabolic heterogeneity within brown adipocyte is an important question for future studies.

This hypothesis is further supported by the effects of chronic insulin treatment on UCP1 expression. UCP1 up-regulation results in an increased thermogenic capacity of brown adipocytes, as reported before [48], and uncoupling of oxidative phosphorylation. As a result, brown adipocytes up-regulate IF1 to prevent unwanted degradation of ATP by ATP synthase. Furthermore, streptozotocin-induced diabetic murine BAT revealed a substantial reduction in UCP1 expression, and insulin treatment of these mice doubled the amount of UCP1 in BAT, suggesting an important role of insulin in UCP1-induced thermogenesis [49]. However, long insulin treatment leads to a selective reduction of mitochondrial respiration and uncoupling in murine BAT [50].

Although Western blotting analyses of the mitochondria-enriched fraction provides the best insight into the insulin-induced changes in the expression of proteins involved in establishing and maintaining the bioenergetic and thermogenic capacity of brown adipocytes, the exact cellular, and mitochondrial localization remains unknown. Given this, we wanted to determine if brown adipocytes displayed the Harlequin effect through the expression of ETC complexes and ATP synthase and used immunofluorescence (IF) to investigate this.

Immunofluorescence analysis revealed the presence of UCP1-positive and UCP1-negative brown adipocytes in BAT, especially in control groups, which is indicative of the Harlequin effect [13]. Moreover, we identified heterogeneity in IF data for ETC complexes, suggesting that different brown adipocytes and different mitochondria within a single brown adipocyte can have different bioenergetic profiles. Co-expression of ETC complexes and parallel changes in their expression patterns following insulin treatment were also observed. Marked differences were apparent in different brown adipocytes, suggesting that insulin modulates the expression of UCP1 and/or ETC complexes. However, IF data showed a more homogenous pattern following chronic high-dose insulin treatment.

This study also suggests that insulin regulates bioenergetic and thermogenic capacities of rat brown adipocytes by modulating the expression of key mitochondrial proteins, i.e., ETC complexes/UCP1. We showed that key bioenergetic proteins (complexes I, II, III, IV, and ATP synthase) and thermogenic protein UCP1 are immunolocalized not only in different brown adipocytes but also in different mitochondrial subpopulations within a single brown adipocyte.

Our results from immunofluorescence and colocalization analysis along with expression and activity of examined complexes strongly suggest the functional heterogeneity of mitochondria in brown adipocytes, which has been proposed previously for other cell types [51,52,53]. The preferential presence of UCP1 or ETC complexes and ATP synthase in mitochondria can be explained by differences in bioenergetic and thermogenic capacity in different brown adipocytes or BAT areas. Some mitochondria contained bioenergetic and thermogenic proteins colocalized in and around lipid droplets, even in white adipocyte-like brown adipocytes, supporting their involvement in metabolic heterogeneity, as showing that peridroplet mitochondria have enhanced bioenergetic capacity [14].

Stoichiometry of the respiratory chain complexes is important for understanding the relative contribution of these complexes to oxidative phosphorylation and is often used as a tool for assessing mitochondrial dysfunction in a cell. Here, we used this tool to assess the overall rate of proton utilization for (a) ATP production by ATP synthase and (b) heat generation by UCP1. The somewhat stable UCP1:IF1 ratio probably indicates that these proteins cooperate to ensure inhibition of ATPase activity, which would be detrimental for normal cell functioning during proton dissipation as heat by UCP1. This could be linked to the coexistence of brown adipocyte subtypes with different thermogenic potentials [54].

Hence, our results suggest that insulin modulates thermogenic and bioenergetic signatures in brown adipocytes by modulating mitochondrial heterogeneity, e.g., mosaicism on the single-cell level. This finding could help to elucidate the role of insulin in energy homeostasis and in various metabolic disorders in which insulin may be the primary cause, such as hyperinsulinemia, insulin resistance, and diabetes. This knowledge, in turn, may lead to novel therapeutic interventions that target BAT. We intend to unravel the detailed molecular mechanisms of insulin-induced cristae remodeling in future studies. Our results also point out that the known Harlequin effect in BAT due to different UCP1 expression among brown adipocytes is a consequence of mitochondrial heterogeneity. Furthermore, BAT Harlequin zonation is a remarkable process by which BAT fulfills its thermogenic/metabolic function and is reflected in the heterogeneity of brown adipocytes and mitochondrial heterogeneity in bioenergetic and thermogenic capacities inside each brown adipocyte.

## 4. Materials and Methods

### 4.1. Experimental Design

All experimental procedures were approved by the Ethics Committee for the Treatment of Experimental Animals (2013/08 from 19 September 2013, University of Belgrade-Faculty of Biology, Serbia). Two-month-old male Wistar rats (190–260 g) were maintained under 22 ± 1 °C, kept in 12 h light/dark cycles with ad libitum access to standard pelleted food and water. The rats were divided into six groups, each consisting of six animals. The first four groups were treated acutely (1 day) or chronically (3 days) with low (0.4 IU/kg) or high dose (4 IU/kg, one intraperitoneal injection per day) of insulin (Mixtard^®^ 30, Novo Nordisk, Bagsværd, Denmark). One IU corresponds to 35 µg of anhydrous insulin. The last two groups served as controls and received an intraperitoneal injection of 0.9% saline for one or three days (1 mL/kg). Three hours after the last injection, animals were sacrificed using a decapitator (Harvard Apparatus, Holliston, MA, USA).

### 4.2. Isolation of Mitochondria-Enriched Fraction

The interscapular BAT was pooled from six rats and placed in an ice-cold 250 mM sucrose medium, freed of white fat and connective tissues, and used for isolation of BAT mitochondria-enriched fraction.

BAT mitochondria-enriched fractions were isolated following a protocol published earlier [55]. Briefly, BAT was minced with fine scissors, homogenized using glass Potter-Elvehjem homogenizer with a Teflon pestle, and filtered through two layers of surgical gauze. All tissues were kept at 0–4 °C during the isolation. Homogenates were centrifuged at 8500× *g* for 10 min at 4 °C in a Beckman Optima L-100 XP ultracentrifuge. After the first centrifugation, the resulting supernatant and fat layer were discarded. The pellet was resuspended in ice-cold 250 mM sucrose, transferred to a clean tube, and centrifuged again at 800× *g* for 10 min. The obtained supernatant was centrifuged at 8500× *g*. The resulting pellet was resuspended in 250 mM sucrose medium with 1% EDTA and 0.6% fatty-acid-free BSA and centrifuged again at 8500× *g* for 10 min. Mitochondria-enriched pellets were resuspended in a minimal volume of 250 mM sucrose medium, and protein concentration was determined using Bradford assay. Aliquots were placed in liquid nitrogen and stored at −80 °C.

### 4.3. Western Blotting

After thawing, protein concentrations of mitochondria-enriched fractions were quantified again using the Bradford assay. Sodium dodecyl sulfate (SDS)–polyacrylamide gel electrophoresis (PAGE) was performed following Laemmli [56]. Ponceau red staining was used to show no differences in total protein quantities (Figure S1). Primary antibodies against: NADH-ubiquinone oxidoreductase subunit NDUFA9 for complex I (2.5 µg/mL, ab55521), subunit A of succinate dehydrogenase SDHA for complex II (0.1 µg/mL, ab14715), ubiquinol-cytochrome *c* reductase core protein II UQCRC2 for complex III (0.5 µg/mL, ab14745), cytochrome *c* (2.5 µg/mL), cytochrome *c* oxidase for complex IV (0.1 µg/mL, ab14744), ATP synthase subunit beta ATPB (1 µg/mL, ab14730), ATPase inhibitory factor 1—IF1 (1 µg/mL, ab110277), and uncoupling protein 1—UCP1 (1 µg/mL, ab10983) were purchased from Abcam (Cambridge, UK). All used antibodies were chosen as they recognize the exact epitope, a small amino acid sequence, which increases their specificity toward the target protein. In a number of previous studies, we tested different antibodies (from various manufacturers) in different tissues and found that the antibodies used here show the best specificity. Immunoreactive bands were visualized using enhanced chemiluminescence and Hyperfilm MP (Amersham), and were quantified using ImageJ software (NIH, Bethesda, MD, USA). The volume represents the sum of all pixel intensities within a band, and 1 pixel = 0.007744 mm^2^. We averaged the ratio of pixels per band for the target protein in corresponding samples from three similar independent experiments. The mean values obtained from the control group were taken as 100%, and those from insulin-treated groups were expressed as percentages against the control.

### 4.4. Determination of Mitochondrial Protein Stoichiometry

Means for western blot densitometry were calculated for each protein by averaging densitometry intensities of three similar independent experiments. Stoichiometries of these proteins were calculated by simply dividing their mean WB densitometry intensities (normalized to 1 µg mitochondrial protein content) with each other.

### 4.5. Immunofluorescence

Immediately after dissection, the left portion of BAT was fixed in 4% paraformaldehyde in 0.1 M phosphate-buffered saline at 4 °C overnight and processed routinely for embedding in paraffin. For immunofluorescence analyses, 7 µm thick paraffin-embedded BAT sections were deparaffinized and rehydrated.

We used a double immunofluorescence assay to analyze the mutual presence of UCP1 and key ETC proteins. After antigen retrieval in 10 mM citrate buffer for 10 min in a microwave oven and washing in 0.1 M Tris-buffered saline (TBS), sections were incubated with 10% normal goat serum (ab7481; Abcam) with 1% BSA for 60 min at room temperature. This was followed by overnight incubation at 4 °C with a mixture of antibodies to UCP1 (1:1000) and NDUFA9 (1:100), UCP1 and SDHA (1:100), UCP1 and UQCRC2 (1:100), UCP1 and complex IV (1:100), UCP1 and ATPB (1:100), UCP1 and IF1 (1:100). After rinsing in TBS with 0.1% Tween-20 (TBS-T), sections were labeled with an appropriate fluorochrome-conjugated secondary antibody mixture. UCP1 was labeled with Alexa Fluor 488 secondary antibody (1:400, A-11034; Life Technologies, Waltham, MA, USA), and other proteins were labeled with Alexa Fluor 633 antibody (1:400, A-21052; Life Technologies). After three washings in TBS-T for 15 min each, the slides were counterstained with nuclear stain Sytox Orange (1:1000, S11368; Life Technologies) for 5 min. As final steps, slides were washed in TBS and mounted with Mowiol.

To analyze the mutual localization of IF1 and ATPB, we used a sequential immunofluorescence assay. Briefly, after antigen retrieval in citrate buffer and blocking with 10% normal goat serum and 1% BSA, sections were incubated overnight at 4 °C with primary antibody to IF1 (1:100). After rinsing in TBS-T, sections were labeled with Alexa Fluor 488 secondary antibody (1:400, A-11029; Life Technologies). Later, the slides were washed three times in TBS-T, blocked with 10% normal goat serum and 1% BSA, and incubated with primary antibody to ATPB (1:100) overnight at 4 °C. Rinsed in TBS-T, sections were incubated with Alexa Fluor 633 secondary antibody (1:400, A-21052; Life Technologies). After washing in TBS-T, sections were counterstained with nuclear stain Sytox Orange (1:1000) for 5 min. In the end, the slides were washed in TBS and mounted with Mowiol.

Images were acquired with Leica TCS SP5 II confocal microscope (Leica Microsystems, Wetzlar, Germany) in sequential mode to avoid crosstalk between channels. This microscopy system consists of an acousto-optical tunable filter (AOTF) for excitation control and acousto-optical beam splitter (AOBS) for excitation-emission separation instead of a dichroic mirror, and the various emission wavelengths are routed to the three filter-free spectral photomultiplier tube (PMT) detectors. The double immunolabeled sections were excited with 488 and 633 nm lasers, respectively. Nuclei were visualized using 543 nm laser, and blue false-colored for clear distinction of green/red channels. The specificity of immunofluorescence was tested by the omission of primary antibodies. To check for nonselective staining (due to primary or secondary antibody specificity), we ran the negative controls in parallel.

### 4.6. Colocalization Analysis

Pearson’s coefficients of correlation were determined using ImageJ plugin Coloc2 at the region of interest (ROI) of randomly localized intramitochondrial regions drawn by freehand and applied to images.

### 4.7. Transmission Electron Microscopy

Aliquots of freshly isolated mitochondria-enriched fractions were fixed with 2.5% glutaraldehyde (*v*/*v*) in 0.1 M Sørensen phosphate buffer (0.1 M Na_2_HPO_4_, 0.1 M NaH_2_PO_4_, pH 7.2). Isolated mitochondria were centrifuged at 14,000× *g* for 10 min. The resulting pellets and BAT were washed in PB and postfixed in 2% osmium tetroxide in the same buffer, then routinely dehydrated using increasing concentrations of ethanol and embedded in Araldite. Ultra-thin sections of isolated mitochondria and BAT were obtained using a Leica UC6 ultramicrotome (Leica Microsystems), mounted on copper grids, and contrasted in uranyl acetate and lead citrate using Leica EM STAIN (Leica Microsystems). Sections were examined on a Philips CM12 transmission electron microscope (Philips/FEI, Eindhoven, The Netherlands) operated at 60 kV and equipped with the digital camera SIS MegaView III (Olympus Soft Imaging Solutions, Münster, Germany). The obtained electron micrographs (pixel size: 8 × 8 nm^2^) were used for confirmation of mitochondrial isolation.

### 4.8. Complex IV Activity Assay

Complex IV activity was measured as the rate of reduced cytochrome *c* oxidation by cytochrome *c* oxidase, as described previously [57]. The reaction buffer was 20 mM potassium phosphate with 15 µM reduced cytochrome *c*, pH 7.4, with the rate of oxidation being monitored by the decrease in absorbance at 550 nm, at 30 °C. The reaction was started by adding 50 µg of mitochondrial protein to 1 mL of the reaction mixture. Cytochrome *c* oxidase activity of treated groups represents the means of three independent experiments and is normalized to an appropriate control, taken as 100%.

### 4.9. ATP Synthase Activity Assay

ATP synthase activity was measured as the rate of hydrolysis of ATP generated by the conversion of phosphoenolpyruvate (PEP) to pyruvate by pyruvate kinase (PK), linked to the reduction of pyruvate to lactate by lactate dehydrogenase (LDH). The reaction buffer was 40 mM Tris-HCl, 10 mM EGTA, 0.2 mM NADH, 2.5 mM PEP, 25 µg/mL Antimycin A, 50 mM MgCl_2_, 0.5 mg/mL LDH, 0.5 mg/mL PK, 2.5 mM ATP, pH 8.0, with the rate being monitored by the oxidation of NADH to NAD+ at 340 nm, at 30 °C. The reaction was started by adding 5 µg of mitochondrial protein to 1 mL of the medium. The ATP synthase activity of treated groups represents the means of three independent experiments and is normalized to an appropriate control, taken as 100%.

### 4.10. Chemicals

All chemicals used in this study were purchased from Sigma-Aldrich (Steinheim, Germany), if not otherwise stated.

### 4.11. Statistical Analysis

ANOVA was used to test within-group comparisons using GraphPad Prism. If the F test indicated an overall difference, Tukey’s test was applied to evaluate the significance of the differences. Statistical significance was set at *p* ≤ 0.05.

## Figures and Tables

**Table 1:** Mitochondrial protein stoichiometry following acute and chronic insulin treatment.

**Acute Treatment**									
	**Control**			**0.4 IU**			**4 IU**		
	ATP synthase	IF1	UCP1	ATP synthase	IF1	UCP1	ATP synthase	IF1	UCP1
IF1	29.97	1	0.80	23.57	1	0.88	25.93	1	0.86
UCP1	37.65	1.26	1	26.88	1.14	1	30.30	1.17	1
**Chronic treatment**									
	**Control**			**0.4 IU**			**4 IU**		
	ATP synthase	IF1	UCP1	ATP synthase	IF1	UCP1	ATP synthase	IF1	UCP1
IF1	26.84	1	0.80	34.55	1	0.84	38.65	1	0.82
UCP1	33.36	1.24	1	41.05	1.19	1	47.10	1.22	1

## Supplementary Materials

The following are available online at https://www.mdpi.com/1422-0067/21/23/9204/s1, Figure S1: Ponceau red staining was used to show no differences in total protein quantities.

## Abbreviations

BAT	Brown adipose tissue
BSA	Bovine Serum Albumin
EDTA	Ethylenediaminetetraacetic acid
ETC	Electron transport chain
IF1	Anti-ATPase inhibitory factor 1
IU	International unit
MEF	Mitochondria-enriched fraction
NADH	Nicotinamide adenine dinucleotide
ROS	Reactive oxygen species
UCP1	Uncoupling protein 1
