# Supplementary material for: Insulin Modulates the Bioenergetic and Thermogenic Capacity of Rat Brown Adipocytes In Vivo by Modulating Mitochondrial Mosaicism
Source: Int J Mol Sci. 2020 Dec 3;21(23):9204. doi: 10.3390/ijms21239204 (PMC7730624; doi:10.3390/ijms21239204)
Supplement: Supplementary file 1 [file ijms-21-09204-s001.pdf]

Supplementary Figure:

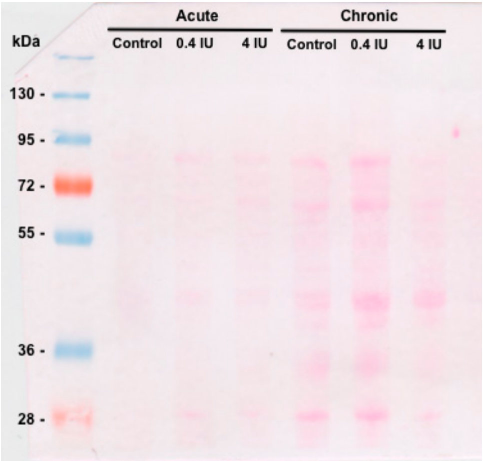

Figure S1. Ponceau red staining was used to show no differences in total protein quantities.
